# Does Binaural Stimulation Enhance Temporal Processing in Young Children?

**DOI:** 10.7759/cureus.40771

**Published:** 2023-06-21

**Authors:** Pradeep Yuvaraj, Krupa S Chacko, Rony Roy, Rahina Abubacker, Aravind Kumar Rajasekaran

**Affiliations:** 1 Speech Pathology and Audiology, National Institute of Mental Health and Neurosciences, Bengaluru, IND

**Keywords:** maximum likelihood procedure, temporal modulation transfer function, monaural temporal processing, binaural temporal processing, gap detection test

## Abstract

Background and objective

Temporal processing abilities help perceive signal changes over time. Efficient temporal processing is necessary for pitch perception, voice identification, and speech perception. It plays a significant role in language development. Internal redundancy of the central auditory nervous plays a role in processing sensory information. There is a need to gain more insights into the maturation of neural hardwiring that supports binaural temporal processing at a young age. The purpose of the present study was to evaluate the difference between monaural and binaural temporal processing in children aged 7-11 years.

Methods

Temporal processing was assessed using gap detection and temporal modulation transfer function (TMTF) tests. The tests were administered in 40 typically developing children with normal clinical auditory sensitivity. The maximum likelihood procedure (MLP), a MATLAB toolbox, was employed to deliver the stimulus. A multivariate analysis followed by post hoc analysis was performed to analyze the data.

Results

There was a significant difference between binaural and monoaural stimulation in children aged 7-11 years. However, there was no statistically significant difference between the right and left ears for gap detection threshold (GDT) and TMTF across all test frequencies.

Conclusion

Based on our findings, binaural stimulation enhances temporal processing in young children.

## Introduction

Speech perception is highly dependent on temporal processing abilities. Temporal processing refers to the auditory system's capability to encode a sound's dynamic durational or temporal-related features within a time interval [1]. Temporal processing is one of the significant clinical indices of central auditory processing [2]. Prosodic elements of speech, such as rhythm, stress, and intonation, which enable the listener to recognize keywords within a phrase and interpret emphases and sarcasm, are difficult for those who are unable to recognize temporal patterns to extract and use [3]. The common tests to measure temporal processing abilities are the gap and modulation detection tests. The gap detection threshold (GDT) refers to the smallest gap between two signals a listener can detect and is expressed in milliseconds (ms). The function of relating the threshold to the modulation rate is known as the temporal modulation transfer function (TMTF). The TMTF test evaluates an individual's ability to detect the presence of amplitude modulation in a sound [4].

The maturation of the auditory pathways occurs in a caudal to rostral direction. The auditory pathway functions are influenced by its maturation [5,6]. Studies have reported that age is a factor in terms of performance in gap detection and TMTF. Children are less sensitive in detecting modulations than adults, showing their temporal processing inefficiencies [7]. In another study, children aged between five and 14 years were compared with adults for their temporal processing ability on TMTF. It was found that the youngest group of children (aged five to six years) were less sensitive to modulation detection than the older children. Furthermore, children even by the age of 14 years were less sensitive to modulation detection than adults. The authors used a bandpass noise carrier to compare the sensitivity to modulation detection of normally hearing children and adults [8]. However, Jain et al. (2015) reported a cut-off age of 10-11 years for achieving adult-like scores [9]. They employed speech and non-speech stimuli and evaluated stop consonant categorization, word recognition, and TMTF. Lister et al. (2011) [10] performed an adaptive clinical test of temporal resolution in a broader age group (7-84 years). They reported that the GDT was higher (right ear: 10 ms; left ear: 8 ms) and highly variable in the youngest group of children (seven to eight years of age) in their study. In contrast, Amaral and Colella-Santos (2010) [11] reported no statistical differences among age groups and ears. They evaluated the temporal resolution skills using the Gaps-in-Noise test in school-aged children aged between eight and 10 years and found no significant differences among age groups and ears. Thus, temporal change detection appears to mature by the second decade of life, while gap detection occurs relatively earlier.

Binaural temporal processing performance in young adults is reportedly better than monaural processing [12]. On amplitude modulation detection thresholds, the slopes of the psychometric function for the monaural and binaural stimuli presentation differed significantly [12]. In young children, the binaural vs. monoaural difference in temporal processing has not been extensively explored. However, this knowledge is essential in clinical setups so that the test results could be compared with age-appropriate references. Furthermore, differences between the binaural and monoaural test scores, if observed, can serve as parameters to gain insights into the maturational aspects. This study examined the difference between monaural and binaural temporal processing in children aged 7-11 years.

## Materials and methods

Selection of participants

Forty children aged 7-11 years balanced for gender ratio were included in the present study. The study setting was a regular school for typically developing children. All the participants underwent routine audiological evaluation (pure tone audiometry, immittance evaluation, and otoacoustic emissions testing). Only children with normal hearing sensitivity (PTA <25dBHL) and speech identification score of more than 90%, normal middle ear functioning, and no history of otological or neurological problems were included. Those with poor academic performance and/or a history of psychological disorders/developmental delay were excluded from the study. Participants fulfilling the inclusion and exclusion criteria were identified as the whole group (7-11 years of age) and further categorized into two subgroups (Group 1: seven to nine years; Group 2: 9.1-11 years). The Institutional Ethics Committee approved the study (MTCS/ISH/No: 5942/2017). Participants' parents consented to the children's participation in the study, and informed consent was sought before the subject's participation. The data was anonymized to keep the individual’s identity confidential. 

Methods of measurement

MATLAB [Matlab code - maximum likelihood procedure (MLP): Grassi & Soranzo, 2009] [13] was used for stimulus generation and recording for both the Gaps-in-Noise test and the TMTF test. The generated stimulus was presented through a clinical audiometer (GSI-61) at a 40 dB sensation level. Tests were done for three conditions: monoaural (right and left separately) and binaural presentations.

Gaps-in-Noise Test

In this test, the participants were instructed to identify a silent interval in the middle of a 750 ms bandpass noise. Depending on the participant's response, the duration of the gap changed dynamically. At the start and end of the gap, the noise had a 0.5 ms cosine ramp. A two-alternative force choice experiment method was used, wherein a standard and a variable stimulus were presented. The variable stimulus had varying gap durations, while the standard stimulus was a wideband noise of 750 ms with no gap.

The test had four sequences, each with 30 stimuli, making it a total of 120 trials. Each trial included a standard stimulus (no gap) and a variable stimulus (two bursts of white noise separated by an interval). The task involved finding the gap in the sequence. The test began by presenting a stimulus with a 64 ms silent period. Following that, the duration of the gap got altered in response to the subject's response (as specified in the MATLAB MLP protocol). Before the test started, subjects received 10 practice presentations. The average GDT in each sequence and across the four sequences can be calculated using the MATLAB procedure.

Temporal Modulation Transfer Function Test

A recurring shift in frequency or amplitude in the signal over time is referred to as temporal modulation. The modulation could be for varied depths as determined by MLP [13]. The amplitude modulation approach was applied in this investigation. The modulation index is a metric for gauging how much an unmodulated carrier's amplitude varies. If it is stated as a percentage, it is referred to as modulation depth (M).

M=(RMS value of a modulating signal)/(RMS value of an unmodulated signal).

Gaussian noise of a duration of 500 milliseconds was sinusoidally amplitude-modulated at 8 Hz, 20 Hz, 60 Hz, and 200 Hz. Gaussian noise was employed in the investigation as it follows the typical amplitude distribution. Depending on the participant's responses, the depth of the modulated signal altered up to an 80% threshold level (as allowed by the MATLAB platform); 20 log10 (M) was the modulation detection threshold in decibels.

Each presentation consisted of two noise stimuli, one of which was modified and presented one following another. A white noise (standard) and a modulated noise (varying) were used as the stimuli, respectively. The task constituted finding the modulated stimuli. There were four sequences, each with 30 stimuli. A modulation detection threshold was obtained after 30 presentations. The modulation detection thresholds for signals with 8 Hz, 20 Hz, 60 Hz, and 200 Hz modulations were obtained using a similar process.

## Results

Forty children aged between 7 and 11 years underwent GDT and TMTF tests. The tests were conducted in binaural and monaural (right and left) conditions. Among 40 children, nine could not perform the Gaps-in-Noise test. The mean scores for GDT (expressed in ms) and TMTF (expressed in dB) for the right, left, and binaural stimulation are presented in Table 1.

**Table 1:** Mean gap detection (ms) and TMTF thresholds (dB) TMTF: temporal modulation transfer function; GDT: gap detection threshold; SD: standard deviation

Test measures	N	Right		Left		Binaural	
		Mean	SD	Mean	SD	Mean	SD
GDT (in ms)	31	5.00	0.53	5.58	0.51	4.177	0.462
TMTF 8Hz (in dB)	40	-13.48	0.75	-12.73	0.71	-17.22	1.09
TMTF 20Hz (in dB)	40	-10.39	0.61	-9.77	0.61	-12.72	0.68
TMTF 60Hz (in dB)	40	-8.17	0.56	-7.52	0.56	-10.53	0.89
TMTF 200Hz (in dB)	40	-6.10	0.45	-5.16	0.31	-7.83	0.47

The GDT and TMTF scores of bilateral, right, and left conditions were compared using the Friedman test followed by post hoc pairwise multiple comparisons with Bonferroni correction. The statistical significance level was set at p<0.05. The binaural perception was significantly different (p<0.001) compared to both monaural conditions (right ear alone and left ear alone presentations), with binaural always being better than monaural conditions (Table 1). However, the right ear and left ear did not show any statistical significance on GDT and TMTF (Table 2).

**Table 2:** Whole-group comparison of binaural, right, and left scores in Gaps-in-Noise and temporal modulation function tests GDT: gap detection threshold; TMTF: temporal modulation transfer function

Test measures	N	Binaural vs. right vs. left		Binaural vs. right		Binaural vs. left		Right vs. left	
		Test statistic	P-value	Test statistic	P-value	Test statistic	P-value	Test statistic	P-value
GDT	31	28.000	<0.001	-0.982	0.020	-1.312	<0.01	-0.656	0.26
TMTF 8Hz	40	57.788	<0.001	-1.188	<0.001	-1.588	<0.01	-0.400	0.22
TMTF 20Hz	40	58.482	<0.001	-1.00	<0.001	-1.588	<0.01	-0.588	0.26
TMTF 60Hz	40	43.956	<0.001	-0.912	<0.001	-1.338	<0.01	-0.425	0.172
TMTF 200Hz	40	49.264	<0.001	-0.988	<0.001	-1.388	<0.01	-0.350	0.353

An analysis was carried out to study if age played a role in the observed binaural processing advantage. The whole group (n=40) was subdivided into two subgroups based on the median age (nine years). Children aged seven to nine years constituted Subgroup 1, and children aged between 9.1 and 11 years constituted Subgroup 2. Table 3 shows that both subgroups showed significant differences (p<0.05) between binaural and monaural stimulation for GDT and TMTF. There was no statistically significant difference between the right and left ear for GDT and TMTF. Friedman test with the statistical significance level set at p<0.05 was used (Table 3).

**Table 3:** Comparison of binaural, right, and left scores within the subgroups GDT: gap detection threshold; TMTF: temporal modulation transfer function

Group	Test measures	N	Binaural vs. right vs. left		Binaural vs. right		Binaural vs. left		Right vs. left	
			Test statistic	P-value	Test statistic	P-value	Test statistic	P-value	Test statistic	P-value
Subgroup 1 (7-9 years)	GDT	15	16.305	<0.001	-0.467	0.604	-1.433	<0.001	-0.967	0.024
	TMTF 8Hz	20	31.444	<0.001	-1.250	0.000	-1.600	<0.001	-0.350	0.805
	TMTF 20Hz	20	26.381	<0.001	-0.900	0.013	-1.425	<0.001	-0.525	0.291
	TMTF 60Hz	20	18.667	<0.001	-0.850	0.022	-1.100	0.002	-0.250	1.00
	TMTF 200Hz	20	21.720	<0.001	-0.825	0.027	-1.125	0.001	-0.300	1.00
Subgroup 2 (9.1-11 years)	GDT	17	13.104	0.001	-0.824	0.049	-1.206	0.001	-0.382	0.795
	TMTF 8Hz	20	26.658	<0.001	-1.125	0.001	-1.575	<0.001	-0.450	0.464
	TMTF 20Hz	20	32.103	<0.001	-1.100	0.002	-1.750	<0.001	-0.650	0.119
	TMTF 60Hz	20	25.595	<0.001	-0.975	0.006	-1.575	<0.001	-0.600	0.173
	TMTF 200Hz	20	27.627	<0.001	-1.150	0.001	-1.550	<0.001	-0.400	0.618

Furthermore, the Mann-Whitney U test (significance level set at p<0.05) was used to study the effect of age within each test condition (binaural, right, and left). For this, the subjects were divided into two subgroups based on the median age (nine years) and compared. There was a significant difference (p<0.05) between the subgroups in terms of both GDT and TMTF (Table 4).

**Table 4:** Comparison of binaural, right, and left scores between the subgroups GDT: gap detection threshold; TMTF: temporal modulation transfer function

Test measures		N	Mann-Whitney U	P-value
GDT	Binaural	32	9.50	<0.001
	Right	32	33.00	<0.001
	Left	32	12.50	<0.001
TMTF 8Hz	Binaural	40	105.50	0.009
	Right	40	69.00	<0.001
	Left	40	56.50	<0.001
TMTF 20Hz	Binaural	40	64.00	<0.001
	Right	40	70.00	<0.001
	Left	40	69.00	<0.001
TMTF 60Hz	Binaural	40	112.50	0.017
	Right	40	100.00	0.006
	Left	40	114.00	0.020
TMTF 200Hz	Binaural	40	69.00	0.000
	Right	40	108.00	0.012
	Left	40	81.00	0.001

## Discussion

Temporal processing is a complex neuronal function that aids in deciphering complex heard auditory signals. The right and left neural hubs in the central auditory pathway are interconnected and can be advantageous for the binaural processing of the incoming signal. Better binaural processing has been reported in adults compared to children. Evaluating the presence or absence of such binaural advantage in younger children may help understand the maturational aspects of the underlying neurophysiological mechanism. Moreover, it could help establish normative data on young children.

In the present study, children performed better in the binaural condition on the temporal processing tests than in the monaural condition for both the whole group and the subgroups (Tables 2, 3). Similar findings have been reported for amplitude modulation in a study of monaural and binaural detection thresholds of amplitude modulation in young adults [12]. An internal (neural) redundancy through binaural interaction along the central auditory pathway should aid better binaural temporal processing. The inferior colliculus (IC), the nucleus of the lateral lemniscus (NLL), and the superior olivary complex (SOC) are the primary sites of binaural contact. The anterior ventral cochlear nucleus (CN), which innervates many SOC subdivisions on both sides of the brain, is where the SOC receives its input [14]. The existence of rigorous functional segregation in these binaural pathways is not well supported, although the analysis of interaural time or level differences (ITD, ILD) appears to be the primary function of certain SOC subdivisions. There is growing evidence to suggest that certain auditory mid-brain regions create topographic representations of auditory space by combining binaural input with spectrum cues from the outer ear. This mechanism adds to the internal redundancy during the binaural temporal processing tasks, thus eliciting better responses. The current study's findings of better performance for binaural temporal processing indicate that the underlying brain mechanism is well-established from an early age.

The results of the present study did not show a statistically significant difference in ear-specific scores (right vs. left) for all the tests (Tables 2, 3). Samelli and Schochat (2008) [15] evaluated the right ear advantage on the gap detection test and concluded that there is no such advantage. Pérez-Pereira et al. (2010) [16] observed similar results, noting no significant difference between the right and left ear scores in the Gaps-in-Noise test. In another study, Carmichael et al. (2008) [17] applied two types of gap detection tasks for three types of contralateral masking conditions. They reported no appreciable differences in thresholds between the ears for either masking or gap detection tests. Balen (1997) [18] studied temporal pattern recognition among 229 typically developing children aged 7-11 years. The author reported that no significant ear difference was noted, even when the test accuracy improved by age for both tests. These results support the findings of the current study. Furthermore, it could be construed based on the results of the present study that there is no difference between the underlying (structural/physiological) right and the left auditory mechanism in processing the temporal signals individually. However, the observed binaural advantage could indicate that a computational factor that combines the right and the left auditory inputs may play a significant role.

In the current study, it is interesting to note a significant difference between the subgroups (Table 4). The results suggest that age is a significant factor in the temporal processing abilities. The improved performance in Subgroup 2 (9.1-11 years) could be attributed to the auditory developmental changes in the central auditory pathway. At younger ages (Subgroup 1), the central auditory system may be less efficient in extracting temporal information [15]. The perceptual abilities directly depend on auditory maturation, and children achieve adult-like performance before/at 10 years of age [16,17,18]. An earlier study reported that modulation detection improved with age [19]. Normal temporal processing is vital for auditory perception, including the perception of speech [20,21]. 

This study has a major limitation: its smaller sample size. It impeded the further subgrouping of subjects to evaluate the effect of age within the selected age range.

## Conclusions

The observed binaural advantage in the present study points to the predisposed capacity of the neural system to process binaural information. Furthermore, maturation is a factor to be considered while evaluating temporal processing in children, and clinicians should use age-appropriate references to interpret the test. Future research could explore the short-term and long-term effects of abnormal binaural temporal processing in children. Also, the effect of early intervention on correctable/treatable hearing conditions could be explored.
